# Ghrelin and Its Analogues, BIM-28131 and BIM-28125, Improve Body Weight and Regulate the Expression of MuRF-1 and MAFbx in a Rat Heart Failure Model

**DOI:** 10.1371/journal.pone.0026865

**Published:** 2011-11-15

**Authors:** Sandra Palus, Robert Schur, Yoshihiro J. Akashi, Barbara Bockmeyer, Rakesh Datta, Heather Halem, Jesse Dong, Michael D. Culler, Volker Adams, Stefan D. Anker, Jochen Springer

**Affiliations:** 1 Applied Cachexia Research, Department of Cardiology, Charité Medical School, Berlin, Germany; 2 Center for Cardiovascular Research, Charité Medical School, Berlin, Germany; 3 Department of Cardiology, Heart Center Leipzig, University Leipzig, Leipzig, Germany; 4 IPSEN, Milford, Massachusetts, United States of America; 5 Centre for Clinical and Basic Research, IRCCS San Raffaele, Rome, Italy; 6 Norwich Medical School, University of East Anglia, Norwich, United Kingdom; Pennington Biomedical Research Center, United States of America

## Abstract

Cardiac cachexia is a serious complication of chronic heart failure with a prevalence of 10–16% and poor prognosis. There are no current therapy options for cardiac cachexia. Ghrelin is the natural ligand for the GHS-1a-receptor and a potential target for conditions associated with cachexia. Ghrelin has been shown to increase weight in several species. The GHS-1a-receptor is not only found in the brain, but also in other tissues, including the myocardium. Human clinical trials with native ghrelin in cardiac cachexia demonstrated increases in appetite, weight and cardiac output.

**Methods:**

Human ghrelin or one of two analogues BIM-28125 and BIM-28131 (also known as RM-131) were tested at 50 nmole/kg/d and 500 nmole/kg/d versus placebo in a rat model of heart failure (myocardial infarction). Animals (SD-rats, approx. 225 g at surgery) received diuretics from day 14 and compounds from day 28 for 4 weeks using osmotic pumps. Weight was monitored and body composition analysed (NMR-scanning). Cardiac function was assessed by echocardiography and hemodynamics.

**Results:**

Animals with MI gained less weight compared to sham rats until start of the therapy (311 g vs 324 g, p = 0.0129). Animals treated with BIM-28131 at 50 nmole/kg/d or all compounds at 500 nmole/kg/d displayed stronger weight gain compared to placebo and sham (all p<0.001). Before treatment, body composition was similar in all groups (average: 36 g fat, 248 g lean). Placebo-treated rats gained no fat, but only lean mass. The active compounds induced both fat and lean mass gain, but to a different extent. The fat-to-muscle-ratio of tissue gain was 0.9±0.07 for BIM-28131 at 50 nmole/kg/d, whereas at 500 nmole/kg/d it was 0.76±0.07 for BIM-28131, 0.68±0.12 for BIM-28125, and 0.48±0.05 for ghrelin. MuRF-1 and MAFbx were differentially regulated by treatment.

**Conclusion:**

Ghrelin is a very promising treatment option for cardiac cachexia, with the analogue BIM-28131 (RM-131) being the most effective compound.

## Introduction

Heart failure is one of the major public health problems, which currently affects over 5 million Americans with 500,000 new cases per year. Clinical trials have shown that beta-blockers [1] and angiotensin-converting enzyme inhibitors [2] reduce the mortality in patients with chronic heart failure (CHF), but CHF still contributes to 250,000 deaths per year. Approximately 15% of the CHF patients display cardiac cachexia, which further impairs the mean survival (50% mortality at 18 months vs. 5 years in non-cachectic patients) [3], [4]. Cardiac cachexia represents a catabolic state, which is characterized by a non-oedematous weight loss of more than 6% over a period of >6 months, which affects not only fat, but also lean mass [5]. The weight loss itself represents a strong independent risk factor for CHF patients [3]. The anabolic growth hormone (GH) and its mediator insulin-like growth factor-1 (IGF-1) are essential for the metabolic homeostasis as well as muscle growth and function. The GH/IGF-1 axis is thought to play a major role in cardiac cachexia, as patients have increased serum GH-levels while IGF-1 is normal or lower [6], [7].

In patients suffering from cardiac cachexia, the alterations of skeletal muscle physiology are associated with a reduction of local IGF-expression [8], while plasma levels appear normal. At the same time, catabolic pathways are activated. Three different pathways are involved in protein degradation of skeletal muscle: lysosomal degradation, calcium-activated proteases, and ATP-dependent and –independent pathways [9], [10]. In particular, the ubiquitin-proteasome pathway (UPS) is vital for the degradation of contractile proteins leading to atrophy if activated inappropriately [11]. A comparison of normal and atrophic rat skeletal muscle showed that two genes were significantly upregulated during the development of atrophy: MuRF1 (for Muscle Ring Finger 1) and atrogin1/MAFbx (for Muscle Atrophy F-box) [12].

Ghrelin is the natural ligand for the GHS-1a receptor and a potential target for treatment of clinical conditions associated with energy balance and cachexia. Administration of ghrelin has been shown to promote increased weight gain and also improve cardiac function in mice, rats, dogs and humans. The GHS-1a receptor and it mRNA are not only restricted to the hypothalamus and the pituitary gland, but are also located in other tissues, including the myocardium. Human clinical trials conducted with native ghrelin in cachexia associated with cardiac cachexia demonstrate increases in appetite, weight and cardiac output without apparent toxicity.

Ghrelin may therefore be an important mediator of metabolic homeostasis and hence the GHSR a potential therapeutic target for cachexia. Aside from metabolic benefits and regulation of feeding behaviour [13], ghrelin has shown some beneficial cardiovascular effects in animal studies [14] as well as human pilot trials [15], making ghrelin an important treatment option for cardiac cachexia.

In this study, two ghrelin-analogues (BIM-28125 and BIM-28131) were tested against human ghrelin in a randomized, placebo-controlled, blinded rat study using the LAD myocardial infarction model. The main focus of this study was the change in body weight and body composition (assessed by NMR-scans) by the treatment of the ghrelin-analogues.

## Methods

### CHF-Model

Myocardial infarction was induced in male Sprague Dawley rats (Harlan-Winkelman, Borchen, Germany) weighing 215 to 230 g by left coronary artery ligation. Control animals underwent a sham operation consisting of a thoracotomy and cardiac exposure without ligation of the coronary artery. Surgery was performed under sterile conditions using ketamin/domitor anesthesia. After surgery, domitor was antagonized with antisedan. All rats were treated with buprenorphin twice daily for two days. The 24 hour mortality rate was 31% and the surviving animals were individually housed and maintained on standard chow with a standard 12-hour light cycle. Starting on day 14 after surgery animals were treated with furosemide (86 mg/L in drinking water). On day 28 the rats were randomized into 7 treatment groups (n = 18 each), which were given either placebo or one of the ghrelin compounds BIM 28125, BIM 28131 or human ghrelin at 50 or 500 nmole/kg/d (all compounds IPSEN Pharmaceuticals) via osmotic mini pumps (Charles River, Sulzfeld, Germany). Pumps were implanted subcutaneously on the back. The sham-group (n = 14) received placebo (Figure S1). Pumps were replaced on day 42 and the animals sacrificed on day 56. Animals with an infarct size of less than 25% of the left ventricle were excluded from this study (n = 5). All procedures have been approved by the local ethics committee (Ladesamt für Gesundheit und Soziales Berlin, Germany; permit number G 0116/05).

### Body composition

Body weight and food intake of the animals were recorded twice per week. Body composition was analyzed by NMR scans (EchoMRI-700, Echo Medical System, Houston, USA) before surgery, on day 28 and on day 56. Measures were fat mass, lean mass, total water and free water content. A mean of 3 measurements per animals was used.

### Echocardiography

Echocardiography was performed prior to sacrifice, using am 15 MHz probe and an Acuson Sequoia system (Siemens, Erlangen, Germany). Anterior and posterior end-diastolic and end-systolic wall thickness, left-ventricular end-diastolic and end-systolic dimensions, ejection fraction as well as fractional shortening were measured.

### Hemodynamics

Hemodynamic studies were performed after 4 weeks of treatment under chloralhydate anesthesia. A polyethylene-50 tubing catheter was inserted via the right carotid artery into the left ventricle for measurement of mean arterial and end-diastolic pressures. Left ventricular contractility (dP/d*t*_max_), end-systolic and end-diastolic pressure were obtained from the ventricular pressure curves, which were converted with a Gould differentiator (G4615). All pressures were registered with a Statham P23 XL transducer and a Gould AMP 4600 amplifier. Heart rate was derived from the arterial blood pressure signal.

### Quantification of MAFbx and MuRF-1 expression

Frozen gastrocenmius samples were homogenized in lysis buffer and protein expression was quantified by Western blot using specific antibodies to MuRF1, MAFbx (generated in rabbits by our group as described [16]). After incubation with a horseradish peroxidase-conjugated secondary antibody, specific bands were visualized by enzymatic chemiluminescence (Super Signal West Pico, Pierce, Bonn, Germany) and densitometry was quantified using a 1D scan software package (Scanalytics, Rockville, USA). Loading differences were controlled by re-probing the blot with an antibody against GAPDH (Hytest, Turku, Finland) [17].

### Statistical Analysis

Numerical values are expressed as mean ± SEM. Comparison of parameters among the 7 groups were made using SPSS 12.0 with a one-way ANOVA, followed by the Tukey post-hoc test. A value of p≤0.05 was considered significant. Comparison of weight gain within one group was calculated with a paired t-test.

## Results

### Animal characteristics

Body weight was similar at the surgery, but infracted animals gained less weight during the first 28 days of the protocol. Body weight at the start of treatment (day 28) was significantly lower in animals receiving infarct surgery (311.8±2.0 g, n = 124) as compared to sham surgery (324.0±4.4 g, n = 14), p = 0.049, 95%CI: −24.2 to −0.2. Infarct size and tibia length were similar between the groups (Table 1).

**Table 1:** Animal characteristics and body weight.

			50 nmole/kg/d			500 nmole/kg/d			ANOVA
	Sham Placebo	Placebo	BIM-28125	BIM-28131	hum ghrelin	BIM-28125	BIM-28131	hum ghrelin	p-values (infarct groups)
Infarct size [%]	-	44.5±2.3	39.7±2.4	42.4±1.9	42±2.2	41.0±2.4	44.4±1.7	44.4±1.9	0.713
Tibia length [cm]	3.94±0.04	3.80±0.03	3.87±0.15	3.93±0.04	3.89±0.03	3.80±0.05	3.83±0.03	3.88±0.03	0.515
Weight at surgery [g]	227.9±2.5	225.0±2.1	227.6±1.6	230.4±2.1	227.4±1.6	225.2±2.3	226.7±1.9	225.6±2.0	0.512
Weight start treatment [g]	322.5±7.4	311.4±5.8	308.8±6.9	324.2±6.9	316.1±3.7	303.2±5.5	313.7±5.6	302.7±6.2	0.053
Weight day 42 [g]	343.3±7.4	338.4±7.6	332.6±6.5	366.3±6.9*	338.8±5.3	353.6±7.7	370.7±8.6**	349.3±8.5	0.002
Weight day 56 [g]	357.8±7.8	339.4±7.5	348.16.4	386.9±8.8***	352.2±5.4	376.6±9.5**	392.3±8.9***	370.9±10.7*	0.001

* : p<0.05,

** : p<0.01,

*** : p<0.001 vs placebo.

### Weight gain

**Figure 1:**
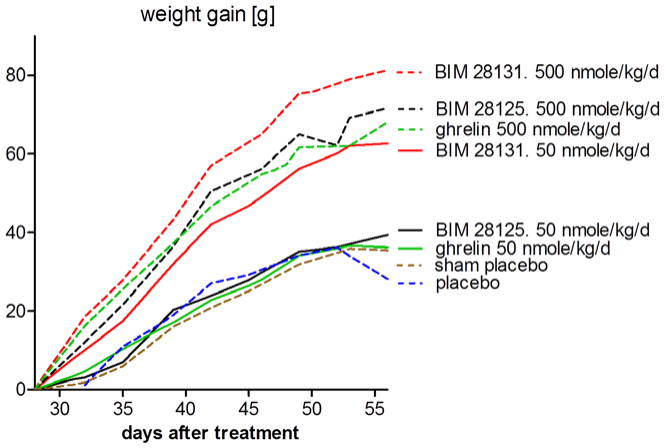
Body weight gain [g] during treatment with ghrelin compounds or placebo (day 28–56). A dose-dependent effect of all compounds was seen. Interestingly, 50 nmole/kg/d BIM-28131 has a higher efficacy than BIM-21125 or human ghrelin at the same dose.

**Figure 2:**
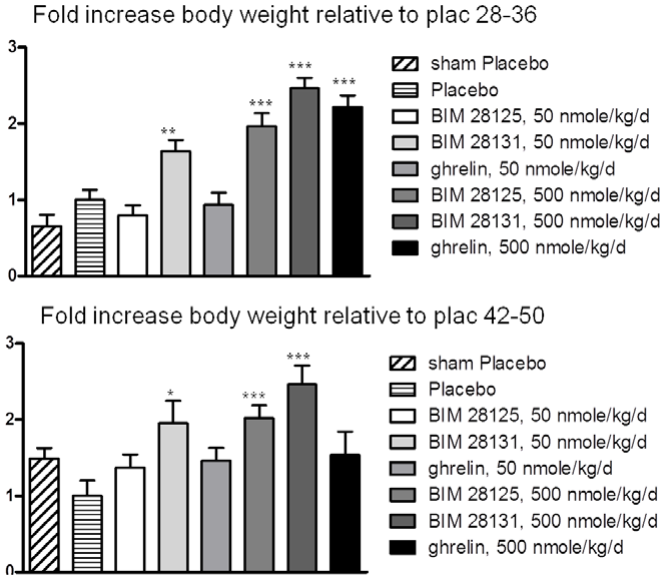
Fold increase of body weight normalised to increase in body weight in the infarct placebo group ( = normal weight gain, i.e. growth) during the first treatment period (day 28–41) and the second treatment period (day 42–56). The first week after pump implantation was chosen for analysis, as the dose/kg becomes lower during treatment due to growth of the animal. Human ghrelin shows tolerance effects, as weight gain during the second period is not significant vs placebo. *: p<0.05, **: p<0.01, ***: p<0.001 compared to placebo.

There was no significantly different increase in body weight in the infarct groups during the first 28 days after surgery (Table 1). On day 42 of the protocol, 2 weeks after starting the treatment with the ghrelin-compounds or placebo, animals receiving either BIM-28131 at 50 nmole/kg/d or BIM 28125, BIM 28131 or human ghrelin at 500 nmole/kg/d showed a significantly stronger weight gain compared to placebo-treated rats and even animals with sham surgery (Table 1, Figure 1). Increasing the BIM 28131 dose 10-times did not result in a significantly higher weight gain compared to the low dose. The weight gain was significantly higher during day 28–36 compared to the weight gain observed day 42–50 in the high dose groups (Figure 2). Interestingly, this effect is not statistically relevant in the low dose groups.

### Food Intake

**Figure 3:**
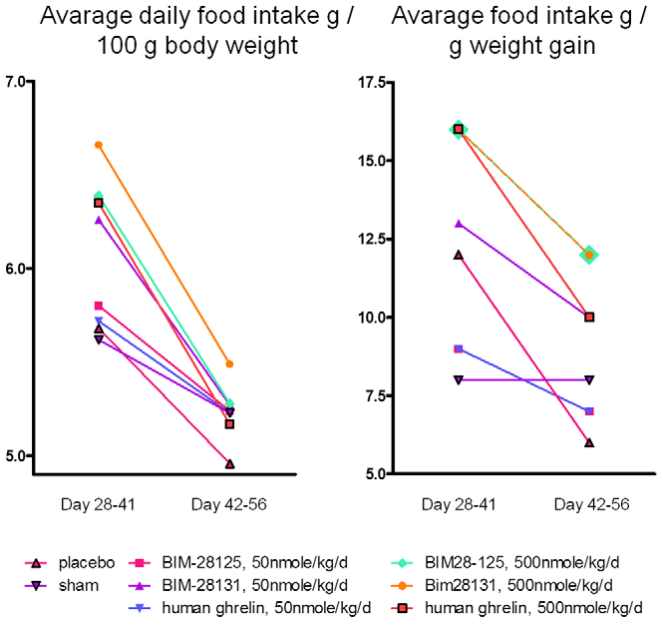
Effect of ghrelin compounds on food intake. A) food intake relative to overall body weight; B) gain of body weight relative to food intake.

Overall the food intake per day adjusted to body weight was getting lower during the 2 treatment periods (period 1: day 28–41, period 2: 42–56). Food intake was induced in all high dose and in the low dose BIM-28131 groups compared to placebo during treatment period 1 (day 28–41, Figure 3), and in the second treatment period all groups given ghrelin compounds had a higher food consumption than placebo (figure 3). Low dose BIM-28131 (6.26±0.07%) was superior to low dose human ghrelin (5.72±10.1%, p<0.001) and low dose BIM-28125 (5.80±0.07%, p<0.001) during the first but not the second treatment period. High dose BIM 28131 (6.66±0.13% and 5.49±0.1 for period 1 and 2 respectively) was superior high dose human ghrelin (6.35±0.12%, p<0.01 and 5.17±0.13%, p<0.01) and BIM-28131 (6.39±0.11%, p<0.01 and 5.28±0.08, p<0.05). While all 3 compounds showed dose dependant effects during the first period (all p<0.001), only BIM-28131 showed a dose-dependent effect during the second treatment period (p<0.05).

### Body composition

**Figure 4:**
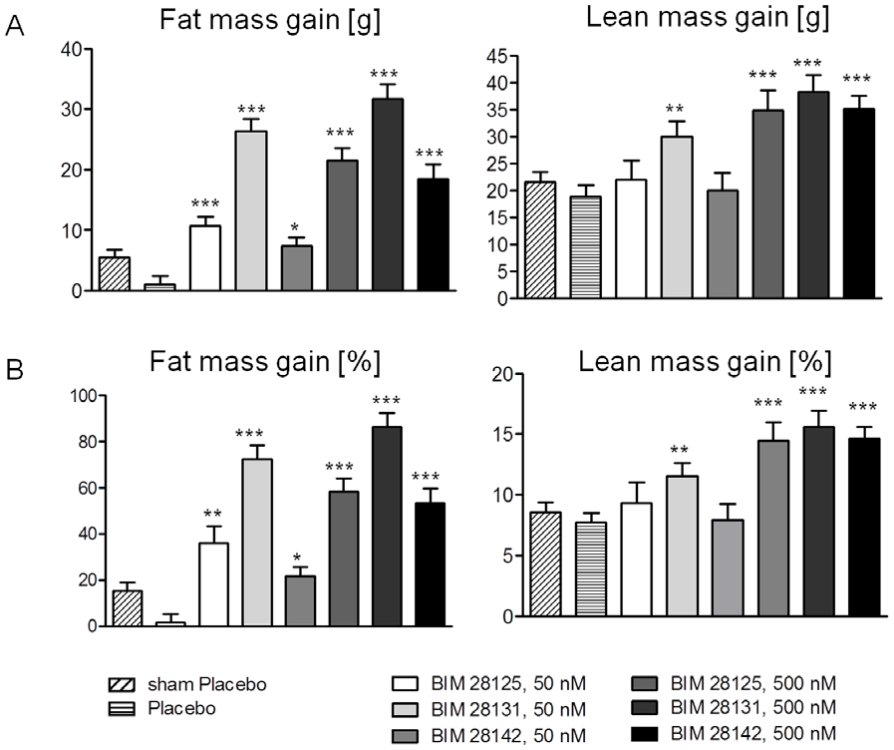
Effect of ghrelin compounds on body composition assessed by NMR scanning. A) absolute gain of fat mass and lean body mass. B) relative gain of fat and lean body mass compared to start of treatment. *: p<0.05, **: p<0.01, ***: p<0.001 compared to placebo.

**Figure 5:**
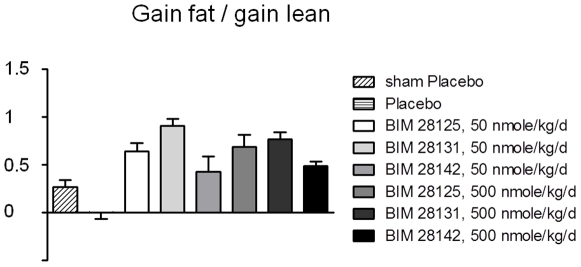
Ratio of fat and lean gain induced by ghrelin compounds. A ratio of 1 means equal fat and lean body mass gain. BIM-28131 (50 nmole/kg/d) has the most benificial ratio of all compounds and doses tested.

The increase in lean and fat mass of the infarct groups did not show any significant difference between surgery and start of treatment (day 28) and the body composition was similar in all groups (average: 36 g fat, 248 g lean). Placebo-treated rats gained no fat, but only lean body mass during the treatment period. The ghrelin compounds induced both fat and lean mass gain, but to a different extent (Figure 4). The ratio of fat to lean gain was 0.90±0.07 for BIM-28131 at 50 nmole/kg/d, while at 500 nmole/kg/d it was 0.76±0.07 for BIM-28131, 0.68±0.12 for BIM-28125, and 0.48±0.05 for native ghrelin (ANOVA: p = 0.0001, figure 5).

### Cardiac function

As expected, the hearts of infarcted animals were significantly heavier than those of sham rats even if they were adjusted to size (tibia length, Table 2). The left ventricle displayed some signs of compensatory hypertrophy (data not shown), resulting in similar weight in sham and infarct rats. The septum weights were higher in all groups treated with ghrelin compounds, except for BIM-28125 at 50 nmole/kg/d (p = 0.073), as compared to placebo or sham animals (ANOVA 0.086). The right ventricle of infarct animals were heavier than sham rats, but displayed no differences within the infarct groups (Table 2).

**Table 2:** heart weight adjusted to size (tibia length).

	Sham Placebo	Placebo	BIM-2812550 nmole/kg/d	BIM-2813150 nmole/kg/d	hum ghrelin50 nmole/kg/d	BIM-28125500 nmole/kg/d	BIM-28131500 nmole/kg/d	hum ghrelin500 nmole/kg/d	ANOVA (infarct groups)
Heart weight [mg]	1055±23	1381±39	1322±36	1454±47	1402±35	1479±45	1509±52	1459±46	0.103
Heart/tibia[mg/cm]	267±5	364±11	342±10	370±12	361±10	384±13	389±14	367±12	0.124
LV/tibia[mg/cm]	132±4	132±3	125±5	127±4	123±4	135±5	127±4	126±5	0.523
RV/tibia[mg/cm]	47±2	98±6	85±7	99±8	95±7	101±9	100±8	94±7	0.695
Sep/tibia[mg/cm]	55±2	55±3	60±2	66±3	62±2	65±3	65±4	62±2	0.086
LA/tibia[mg/cm]	7.7±0.5	23.9±1.8	21.8±2.2	23.3±1.6	23.8±1.7	21.6±1.9	28.9±2.4	26.5±2.5	0.077
RA/tibia[mg/cm]	8.7±0.5	19.3±2.3	16.9±1.6	19.0±1.9	18.8±2.2	22.4±1.9	22.1±1.9	21.1±2.1	0.260

LV: left ventricle, RV: right ventricle, Sep: Septum, LA: left atrium, RA: right atrium.

**Figure 6:**
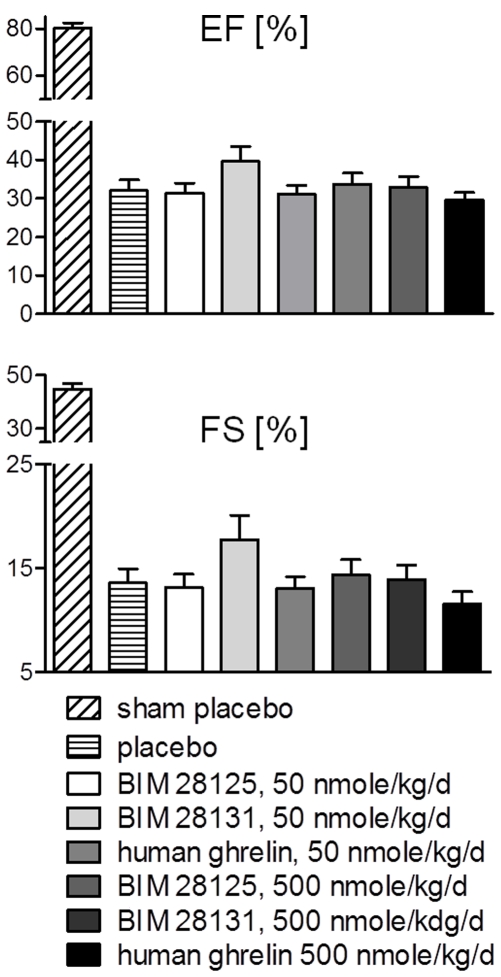
Ghrelin compounds have no effect on cardiac function. A small, non-significant improvement of left ventricular (LV) ejection fraction (EF) and fractional shortening (FS) was seen in the 50 nmole/kg/d BIM-28131 group compared to placebo.

Cardiac function was analysed with echocardiography and hemodynamics (Table S1). Overall no beneficial effect of treatment with ghrelin analogues was seen. However, a small, non-significant improvement of BIM-28131 was observed in the ejection fraction (p = 0.127) and fractional shortening (p = 0.137, Figure 6).

### Expression of MAFbx and MuRF-1

**Figure 7:**
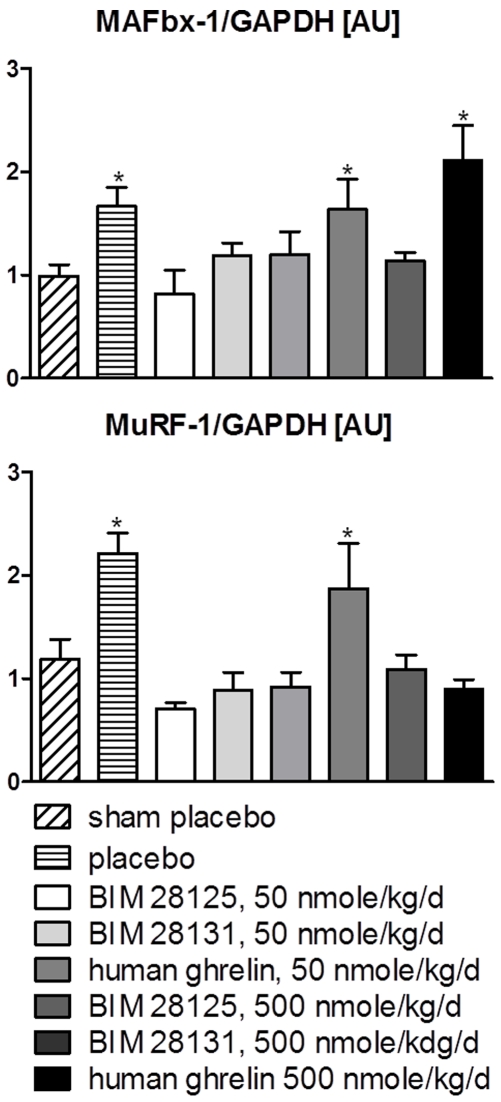
Induction of E-3 ubiquitin ligases MuRF-1 and MAFbx in gastrocnemius muscle by myocardial infarction. Low dose of the compounds reduced MuRF-1 and MAFbx expression. Treatment with high dose BIM-28131 and human ghrelin reduced MuRF-1 (A) and only treatment with high does BIM-28131 reduced MAFbx (B). *: p<0.05 vs sham.

Both E3-ligases which are thought to be the rate limiting step of the ubiquitin-protesome system, were up-regulated in gastrocnemius muscle by myocardial infarction (Figure 7). Expression of MAFbx was reduced to normal levels by 50 nmole/kd/d BIM-28125 and 500 nmole/kg/d BIM-28131 only (Figure 7 A), while MuRF-1 was down-regulated to sham levels by all compounds with the exception of the 500 nmole/kg/d BIM-28125 group (Figure 7 B).

## Discussion

A therapeutic role for the peptide hormone ghrelin has been proposed in patients suffering from end-stage heart failure and cardiac cachexia [15]. Ghrelin has been shown to increase appetite and body weight, and it is thought to have beneficial effects on cardiac function [14], [15] [18]. The increase in food intake is thought to be independent of the GHS-receptor [19]. While Nagaya et al. [14] have shown weight and cardiac effects on rat ghrelin, they have not assessed the body composition changes of treatment. The present study was designed to characterize the effects and the efficacy of two peptide ghrelin analogues (BIM 28125 and BIM-28131) compared to native human ghrelin (BIM-28142), which only differs from rat ghrelin by two residues and shows the same pharmacological activity on the rat receptor as the rat peptide [20].

This study demonstrates that all three compounds at the 500 nmole/kg/d and also BIM-28131 at the 50 nmole/kg/d induce weight gain superior to that of the placebo and even of non-infarcted animals treated with placebo. A beneficial effect of ghrelin on body weight has also been described in human and rat [21] [22]. The overall weight gain significantly decreases in all high dose groups with time. This is a quite physiological phenomenon, because the growth rate of the animals slows, as the animals get older. However when the data is adjusted to the gain seen in the placebo group taken as baseline growth/gain, it is remarkable that human ghrelin actually shows strong tolerance effects compared to BIM-28125 and BIM-28131, which keeps its potency (approx. 2.5 fold increase in body weight gain vs placebo in both time periods).

While the placebo treated infarcted animals could not built up energy reserves, i.e. fat, and only gained lean mass, the animals treated with ghrelin compounds gained fat and lean mass. Contrary to earlier reports [23] human ghrelin induced more lean than fat gain, whereas the ratio of gains seen in animals receiving BIM-28131 was balanced, which may reflect differences in the affinity of the compounds to the GHSR type 1a and typ 1b [24]. Moreover, our study showed an increase of MuRF-1 and MAFbx protein levels in gastrocnemius muscle of rats with myocardial infarction compared to sham surgery, which has also been described for the myocardium [16]. This was reversed in animals treated with all three ghrelin compounds at low dose and from the BIM-28131 high dose group. This likely reflects higher activity of the UPS in skeletal muscle of infracted rats, which is normalized by treatment with ghrelin compounds. A similar finding of a ghrelin receptor agonist has been reported in a severe burns model [25].

The increase in fat mass may have important consequences in the therapy of cardiac cachexia patients, as it has been shown recently that the fat content is inverse related to survival in hemodialysis patients [26]. This effects has also been observed in approx. 500 chronic heart failure patients, where the 12-month mortality was highest (14%) in the low fat mass quartile (4–21%) and lowest (3%) in the high fat mass quartile (>30%) [27]. Also a reduction of major clinical events has been reported in CHF patients with a BMI>30 group and even stronger in the high fat group [28]. Lean mass gain on the other hand can be seen as an improvement of the quality of life, as more lean mass, i.e. muscle, is bound to lead to a greater physical capacity [29]. Hence, the ideal cachexia treatment should aim at raising both, fat and lean mass, which all three compounds used in this study are capable of, but only BIM-28131 shows this effect in the low dose and induces a more balanced gain of fat and lean mass. Using a lower dose in a clinical setting potentially reduces side effects and is therefore favourable.

Previous studies have shown that ghrelin induces food intake [30] by activating neuropeptide Y neurons in the hypothalamic arcuate nucleus [31], an effect that has also been observed in CHF patients after intravenous administration of ghrelin [15]. Contrary to those reports a 4-day rat study did not find an increased food intake but still found an increased body weight [32]. This study shows an increase in food consumption in all high dose groups, as well as the low dose BIM-28131 group compared to placebo. Generally, the food intake was declining during the study, but that may be accounted for by the physiological slowing growth rate of the young animals.

Previous reports have suggested a strong clinical improvement of cardiac function by ghrelin in, both, human and animal studies [14] [15]. In these previous studies human ghrelin significantly increased cardiac output, ejection fraction and fractional shortening and decreased systemic vascular resistance. In the small, open-label human trial few side effects were observed [15]. In our study, however, no beneficial effects of ghrelin compounds were seen on heart function. Although a positive trend was observed in ejection fraction and fractional shortening for BIM 28131. This may be due to the continuous infusion of the compounds in our study compared to the three times daily injection used in the other studies, which would produce peaks of compounds in plasma. These peaks may have differential effects on downstream targets compared to a steady compound level.

Conclusion: Ghrelin and related analogues are potent inducers of weight gain in a heart failure situation. BIM-28131 is superior to BIM-28125 and human ghrelin in weight gain and induces a balanced gain of fat and lean tissue, while normalizing the expression of MuRF-1 and MAFbx. This may make BIM-28131 an ideal candidate for the development of a novel therapy strategy to treat severe heart failure and cardiac cachexia.

## Supporting Information

Figure S1 Schematic overview of the study design.
(TIF)

Table S1 Heart function assessed by echocardiography and invasive hemodynamics. No significant effect of treatment with ghrelin or ghrelin analouges on cardiac function was observed.
(DOCX)
