# Supplementary figures and images for: Ghrelin and Its Analogues, BIM-28131 and BIM-28125, Improve Body Weight and Regulate the Expression of MuRF-1 and MAFbx in a Rat Heart Failure Model
Source: PLoS One. 2011 Nov 15;6(11):e26865. doi: 10.1371/journal.pone.0026865 (PMC3216926; doi:10.1371/journal.pone.0026865)

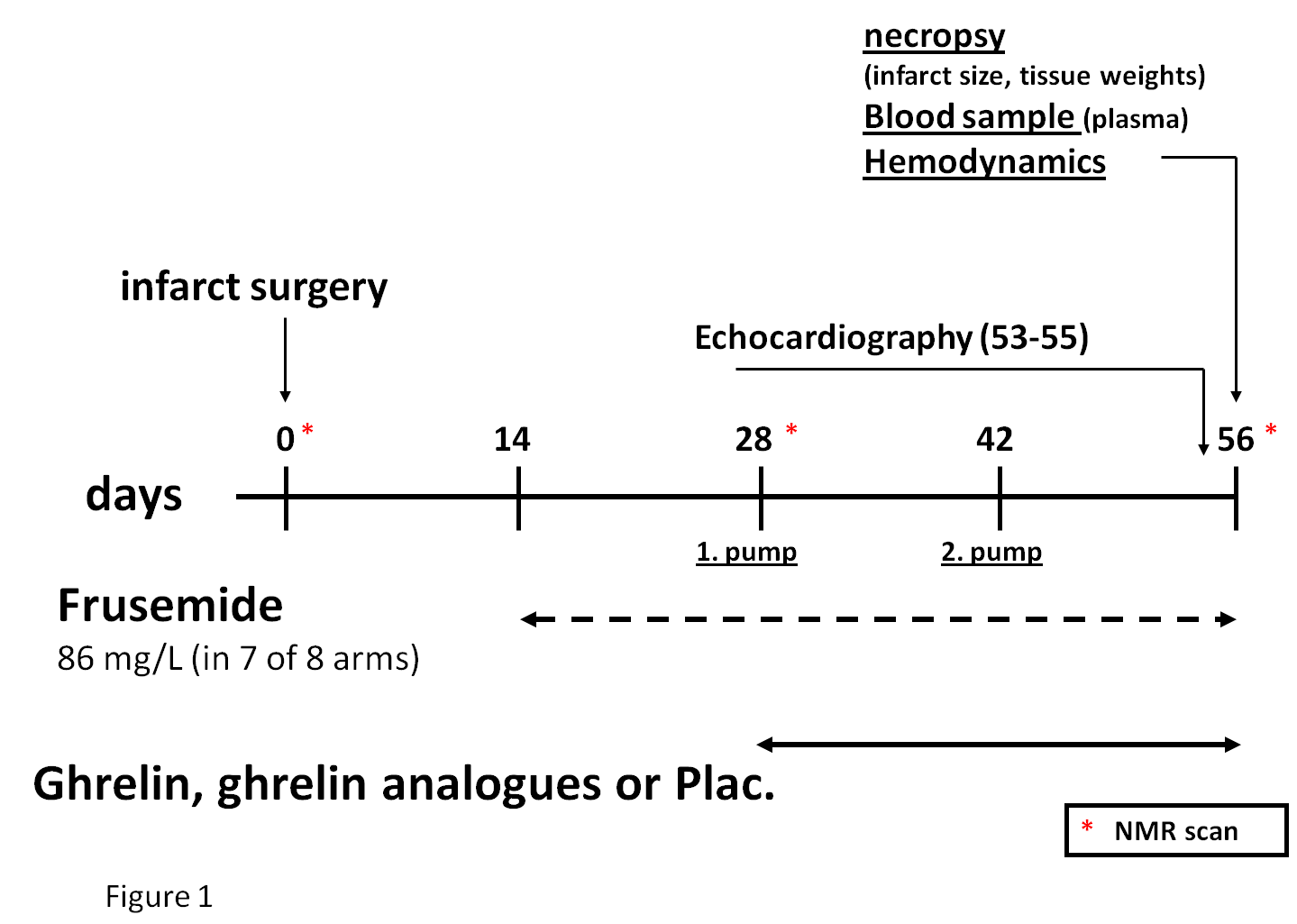

Supplement: Figure S1 — Schematic overview of the study design. (TIF) [file pone.0026865.s001.tif]
