# Supplementary material for: Ghrelin and Its Analogues, BIM-28131 and BIM-28125, Improve Body Weight and Regulate the Expression of MuRF-1 and MAFbx in a Rat Heart Failure Model
Source: PLoS One. 2011 Nov 15;6(11):e26865. doi: 10.1371/journal.pone.0026865 (PMC3216926; doi:10.1371/journal.pone.0026865)
Supplement: Table S1 — Heart function assessed by echocardiography and invasive hemodynamics. No significant effect of treatment with ghrelin or ghrelin analouges on cardiac function was observed. (DOCX) [file pone.0026865.s002.docx]

|  | **BIM-28125**  **50nmole/kg/d** | **BIM-28131**  **50nmole/kg/d** | **hum ghrelin 50nmole/kg/d** | **BIM-28125**  **500nmole/kg/d** | **BIM-28131**  **500nmole/kg/d** | **hum ghrelin 500nmole/kg/d** | **Placebo** | **Sham Placebo** | **ANOVA (infarct groups)** |
| --- | --- | --- | --- | --- | --- | --- | --- | --- | --- |
| E/A | 1.07 ± 0.06 | 1.77 ± 032 | 1.92 ± 0.35 | 1.58 ± 0.28 | 2.07 ± 0.21 | 2.86 ± 0.97 | 2.01 ± 0.21 | 1.13 ± 0.03 | 0.065 |
| LVESD cm | 0.896 ± 0.023 | 0.857 ± 0.039 | 0.929 ± 0.018 | 0.923 ± 0.029 | 0.929 ± 0.023 | 0.949 ± 0.026 | 0.941 ± 0.022 | 0.435 ± 0.025 | 0.296 |
| LVEVD cm | 1.025 ± 0.026 | 1.033 ± 0.027 | 1.069 ± 0.019 | 1.070 ± 0.025 | 1.079 ± 0.018 | 1.082 ± 0.028 | 1.089 ± 0.018 | 0.782 ± 0.21 | 0.351 |
| PWTD cm | 0.163 ± 0.005 | 0.169 ± 0.004 | 0.157 ± 0.004 | 0.156 ± 0.011 | 0.156 ± 0.008 | 0.153 ± 0.008 | 0.148 ± 0.009 | 0.176 ± 0.009 | 0.154 |
| PWTS cm | 0.207 ± 0.009 | 0.244 ± 0.010 | 0.222 ± 0.009 | 0.206 ± 0.014 | 0.217 ± 0.011 | 0.201 ± 0.010 | 0.212 ± 0.014 | 0.313 ± 0.013 | 0.097 |
| PWTT % | 27.9 ± 5.0 | 44.3 ± 5.8 | 42.8 ± 5.6 | 33.5 ± 6.2 | 39.3 ± 5.1 | 24.6 ± 10.3 | 44.0 ± 5.6 | 82.9 ± 10.6 | 0.167 |
| HR bpm | 482 ± 12 | 460 ± 16 | 471 ± 9 | 454 ± 14 | 444 ± 21 | 489 ± 12 | 484 ± 12 | 441 ± 9 | 0.254 |
| sBP mmHg | 95 ± 3 | 90 ± 3 | 88 ± 2 | 91 ± 3 | 81 ± 5 | 89 ± 3 | 86 ± 3 | 117 ± 2 | 0.112 |
| dBP mmHg | 65 ± 3 | 62 ± 4 | 91 ± 3 | 64 ± 2 | 54 ± 4 | 61 ± 3 | 61 ± 3 | 81 ± 2 | 0.386 |
| dt/dP_max_ | 3132 ± 228 | 2687 ± 181 | 2874 ± 170 | 2870 ± 188 | 2436 ± 239 | 2785 ± 144 | 3069 ± 171 | 4964 ± 102 | 0.267 |
| dt/dP_min_ | -2856 ± 239 | -2443 ± 162 | -2642 ± 195 | -2707 ± 197 | -2205 ± 209 | -2603 ± 125 | -2474 ± 154 | -5312 ± 79 | 0.228 |

Supplemental table: heart function assessed by echocardiography and invasive hemodynamics. No significant effect of treatment with ghrelin or ghrelin analouges on cardiac function was observed.
